# Spatial Patterns and Natural Recruitment of Native Shrubs in a Semi-arid Sandy Land

**DOI:** 10.1371/journal.pone.0058331

**Published:** 2013-03-07

**Authors:** Bo Wu, Hongxiao Yang

**Affiliations:** 1 Institute of Desertification Studies, Chinese Academy of Forestry, Haidian, Beijing, China; 2 College of Resources and Environment, Qingdao Agricultural University, Chengyang, Qingdao, Shandong Province, China; Cirad, France

## Abstract

Passive restoration depending on native shrubs is an attractive approach for restoring desertified landscapes in semi-arid sandy regions. We sought to understand the relationships between spatial patterns of native shrubs and their survival ability in sandy environments. Furthermore, we applied our results to better understand whether passive restoration is feasible for desertified landscapes in semi-arid sandy regions. The study was conducted in the semi-arid Mu Us sandy land of northern China with the native shrub *Artemisia ordosica*. We analyzed population structures and patterns of *A. ordosica* at the edges and centers of land patches where sand was stabilized by *A. ordosica*-dominated vegetation. Saplings were more aggregated than adults, and both were more aggregated at the patch edges than at the patch centers. At the patch edges, spatial association of the saplings with the adults was mostly positive at distances 0.3–6.6 m, and turned from positive to neutral, and even negative, at other distances. At the patch centers, the saplings were spaced almost randomly around the adults, and their distances from the adults did not seem to affect their locations. A greater number of *A. ordosica* individuals emerged at the patch edges than at the patch centers. Such patterns may have resulted from their integrative adjustment to specific conditions of soil water supply and sand drift intensity. These findings suggest that in semi-arid sandy regions, native shrubs that are well-adapted to local environments may serve as low-cost and competent ecological engineers that can promote the passive restoration of surrounding patches of mobile sandy land.

## Introduction

Land desertification taking place in semi-arid sandy regions has many detrimental effects. Landscapes were deprived of vegetation as a result of desertification, thereby exposing sand to potential wind forces. Strong winds can push sand into drifting on the ground, and even occasionally generate sand storms [1], [2]. Some dunes become destabilized, and may shift from one place to another. The Mu Us sandy land in semi-arid regions of northern China is characterized by such conditions [2], [3]. From the 1950s to the 1990s, vegetation of many landscape patches, where sand was formerly well-stabilized, suffered extensive damage. During this period, the area of bare sand increased from 30.24% to 44.53% of the total area of Mu Us, and the area of semi-bare sand increased from 10.89% to 21.44% [3]. Local residents took great efforts to restore the landscape. Living vegetation is considered the best materials to cover and stabilize sand, being effective, persistent and low-cost [4].

In the Mu Us region, landscape patches mainly belong to stabilized sand land (SSL) and mobile sand land (MSL) [5]. SSL patches are normally covered by well-developed vegetation that features short shrubs and grasses. Because of such vegetation, sand is well stabilized and unable to drift with winds. MSL patches are not covered by vegetation, and sand gets ready to drift with winds. SSL and MSL patches are connected in a mosaic-like pattern with transitional belts where vegetation is sparse and sand is semi-stabilized. The local people have undertaken many projects to stabilize and restore the MSL patches [6], [7]. However, fierce sand drifting could disable plant establishment, so that bitterly hindered these projects [4], [8].

A variety of artificial barriers have been used to stabilize bare sand in MSL patches, so as to facilitate plant establishment and vegetation restoration [4], [9], [10]. Approaches that require human intervention are classified as active ecological restoration. This type of restoration usually requires high financial costs and significant human resources. In areas characterized by severe and extensive desertification, people are typically too impoverished to afford such costs. Passive ecological restoration entirely depends on natural establishment and development of plant communities, so that requires no human intervention and expenses. The use of passive restoration has been criticized because of the length of time required, and works only if people have enough patience and time to wait for success [4], [11], [12]. Even so, passive restoration is an attractive option for local residents who are already impoverished but may be better off by taking advantages of passive restoration, instead of exclusively depending on active restoration. We assumed that some native shrubs that grow in SSL patches may be able to promote passive restoration of surrounding MSL patches. To explore this perspective, we selected the Mu Us sandy land and its native shrubs of *A. ordosica* Krasch for this study.

As a native species, *A. ordosica* has adapted well to natural conditions of the Mu Us sandy land [13], [14]. In many SSL patches, *A. ordosica* is often the most dominant species. Local people choose to plant this species in MSL patches to stabilize sand, and they have found that many other grasses and herbs could follow to settle and proliferate [15], [16]. The present study was designed to investigate population structures and spatial patterns of *A. ordosica* because they are visible surrogates of intangible population processes [17], [18], [19]. More generally, our study contributes to a better understanding of landscape restoration in the Mu Us region [20]. The following questions are addressed: 1) how surviving *A. ordosica* shrubs are spatially organized, 2) how *A. ordosica* populations recruit, and 3) whether passive restoration is feasible for the desertified Mu Us region.

## Materials and Methods

### Study Site and Materials

**Figure 1:**
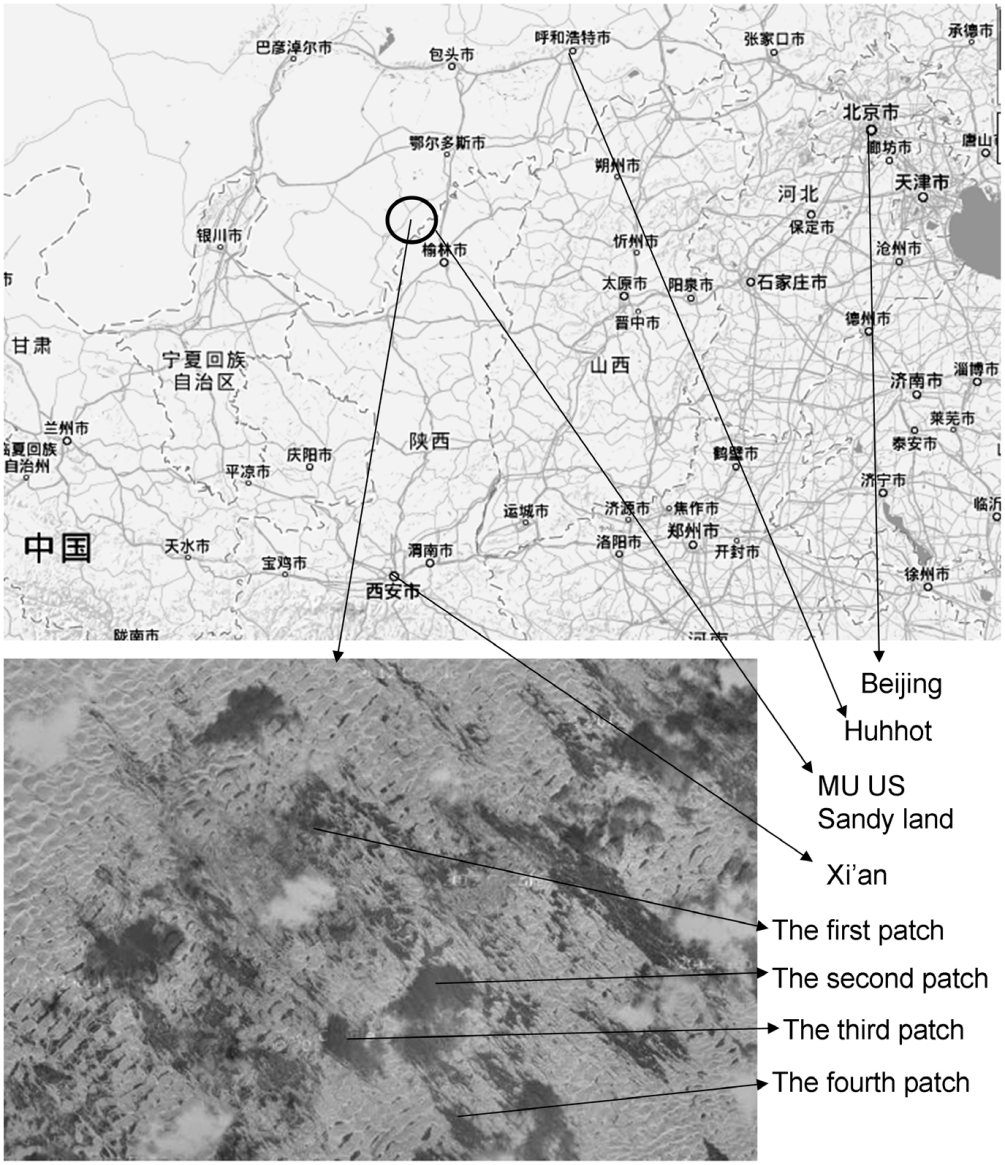
The location of the Mu Us sandy land in northern China. The upper larger map indicates the location of the Mu Us sandy land, and the lower smaller satellite map indicates the four investigated *A. ordosica* patches, which were surrounded by bare mobile sand.

The Mu Us sandy land is located in northern China, and it connects the Mongolia and Loess Plateaus (107°20′ to 111°30′ E, and 37°27′ to 39°22′ N, Figure 1). Elevations vary between 1,200 m and 1,600 m. The sand is deep and extensive. Annual precipitation normally ranges from 250 mm to 450 mm, 60% to 80% of which occurs from June to August. During 49–60 days per year, winds blow faster than 22.7 km/h, the threshold velocity that can cause sand drifting [21]. Annual mean temperatures are approximately 6–8.5°C, with a monthly mean temperature of 22°C in July and −11°C in January. Such conditions are somewhat adverse to plant growth and vegetation development [4], [22], [23]. Several decades prior to the year 2000, human disturbances such as overgrazing, irresponsible farming activities, and arbitrary shrub cutting caused serious landscape desertification [5], [24]. Many SSL patches which were formerly large and widespread became fragmented and replaced by larger MSL patches.

Prior to desertification, original vegetation of SSL patches was mainly dominated by *A. ordosica*, a low-growing shrub species. *Artemisia ordosica* individuals usually do not grow taller than 100 cm and have many paratactic branches that converge beneath or near the ground [13], [25]. Individual shrubs produce a large quantity of tiny seeds with 0.311 g of 1000-grain-weight, which are susceptible to airborne dispersal over several kilometers [26]. Once this species successfully colonizes bare sand, other grasses and herbs will likely follow to colonize, such as *Cleistogenes squarrosa*, *Oxytropis psammocharis*, *Lespedeza davurica*, *Astragalus melilotoides*, *Allium mongolium*, *Inula salsoloides*, and *Agropyron mongolicum* [13].

### Field Surveys

In August 2008, we investigated SSL patches via four samples, where vegetation was still dominated by *A. ordosica* (Figure 1). All the sample patches were approximately larger than 1 km^2^ and surrounded by bare mobile sand. The patches included some low dunes about 2 m high and inter-dune plains about tens of meters broad. The distances between the patches were more than 2 km. We surveyed the four patches through a total of eight 30 m×30 m plots. Two plots were assigned to a patch. One was established at the patch edge and the other near the patch center. In the plots, we recorded information for every *A. ordosica* individual, including narrow (*n*) and wide (*w*) crown-diameters, height (*h*), and relative position in the pertinent plot. We divided each of the plots into many consecutive sub-plots (2 m×2 m), and within each sub-plot we measured the shrub spatial positions with an accuracy of approximately 1 cm. These plots were almost flat and initial conditions prior to the appearing of vegetation was likely homogeneous, although conditions later changed with vegetation development [27], [28]. No other shrubs were present except *A. ordosica*. The coverage and height of the grasses and herbs were much lower than those of *A. ordosica*, so that their influences on *A. ordosica* and the environments were assumed to be negligible.

No specific permits were required for the described field studies. No specific permissions were required for these locations/activities. The location is not privately owned or protected in any way. The field studies did not involve endangered or protected species.

### Data Analysis

Younger *A. ordosica* shrubs usually have smaller crowns and shorter heights than the older individuals. The variables *n*, *w*, and *h* showed positive correlation and minor differences. Thus, all individuals were divided into two groups, namely, adults and saplings, by comparing their averages of *n*, *w* and *h*. An individual with an average of less than 30 cm was considered a sapling; otherwise, it was classified as an adult.

We calculated the coverage (*cov*) of *A. ordosica* in each plot, which was the total area of all *A. ordosica* crowns divided by plot area
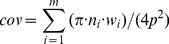
(1)where *cov* refers to *A. ordosica* coverage of a plot, *n_i_* and *w_i_* respectively refer to narrow and wide crown-diameters of the *i-*th individual, *m* is the total number of all individuals that appeared in the same plot, and *p* refers to plot side length. The proportions of adults and saplings to their total number in each plot were also calculated, providing insights into population structure. The T-test method was used to check whether coverage and population structure differed between the patch edges and the patch centers.

We then analyzed shrub spatial patterns using the O-ring statistic, a non-cumulative second order spatial statistic that evaluates the expected number of points at increasing distance (*r*) from a given point in a pattern [29]. The univariate O-ring statistic (*O_11_*_(*r*)_) was used to detect whether adult shrubs exhibited a tendency to aggregate, and the distance (*r*) was defined to vary from 0 m to 15 m with an incremental interval of 0.3 m. In view of the heights of the shrubs, this range of distances was appropriate since shrubs can affect wind speeds in a vicinity up to ca.10-times of their heights [30], [31]. The same analyses were performed also for shrub saplings. Implementation of second-order spatial statistics such as the O-ring requires the proper selection of null models. In these analyses, the null models were that all individuals followed heterogeneous Poisson distribution, which removes false aggregation caused by first-order density. By comparing with the null model, we could judge how shrubs affect surrounding conditions and neighboring shrubs. We used the bivariate O-ring statistic (*O_12_*_(*r*)_) to analyze the spatial association of saplings relative to adult shrubs, using the same range of lag distances (0–15 m) with an incremental interval of 0.3 m. For these analyses, the null models were that all adults were position-fixed and all saplings randomly spaced relative to adults. That is, the null hypothesis was that spatial locations of saplings were not influenced by the locations of adults. Programita, a suitable software package for point pattern analysis, was used to conduct the O-ring analyses [29]. This software calculates such statistics with a grid-based numeric approach, which deals with the problem of edge correction satisfactorily.

Programita was also used to estimate 95% simulation envelopes for both univariate and bivariate O-ring statistics [29]. We performed 199 simulations and selected the 10^th^ highest and lowest values to build the upper and lower envelopes. When the actual *O_11_*_(*r*)_ statistics were higher than their estimated upper envelopes, shrubs were significantly aggregated [29], [32], [33]. When the actual statistics were below their estimated lower envelopes, shrubs were significantly dispersed. Values of the actual statistics between the estimated upper and lower envelopes meant that shrubs were randomly distributed. In the case of bivariate analyses, values of *O_12_*_(*r*)_ above the estimated upper envelopes indicate that saplings were positively associated with adults [29], [32], [34]. Values of *O_12_*_(*r*)_ below the estimated lower envelopes indicate that saplings were negatively associated with adults, and values of *O_12_*_(*r*)_ between the estimated upper and lower envelopes indicate that saplings were neutrally associated with adults.

## Results

### Structure of *A. ordosica* Populations

At the patch edges, *A. ordosica* populations exhibited lower coverage (10% to 15%), higher sapling proportion (30% to 60%), and lower adult proportion (40% to 70%) than those of the patch centers (Table 1). At the patch centers, *A. ordosica* populations exhibited the opposite conditions: higher coverage (40% to 50%), lower sapling proportion (2% to 5%), and higher adult proportion (>95%) (Table 1).

**Table 1:** Coverage and population structure of *A. ordosica* in the four patches.

	Coverage	Individuals in the 900-m^2^ plots		
		Total	Saplings (proportion)	Adults (proportion)
	11.2%	865	515 (59.5%)	350 (40.5%)
Patch edges	13.8%	594	244 (41.1%)	350 (58.9%)
	15.1%	839	493 (58.8%)	346 (41.2%)
	15.3%	426	116 (27.2%)	310 (72.8%)
	47.3%	900	23 (2.6%)	877 (97.4%)
Patch centers	40.2%	745	36 (4.8%)	709 (95.2%)
	41.9%	859	22 (2.6%)	837 (97.4%)
	39.4%	925	44 (4.8%)	881 (95.2%)
Probability for equality	<0.001*		0.012*	0.012*

Note: *indicates a significance level of *p*<0.05 (by T-test).

### Spatial Patterns of *A. ordosica* Individuals

**Figure 2:**
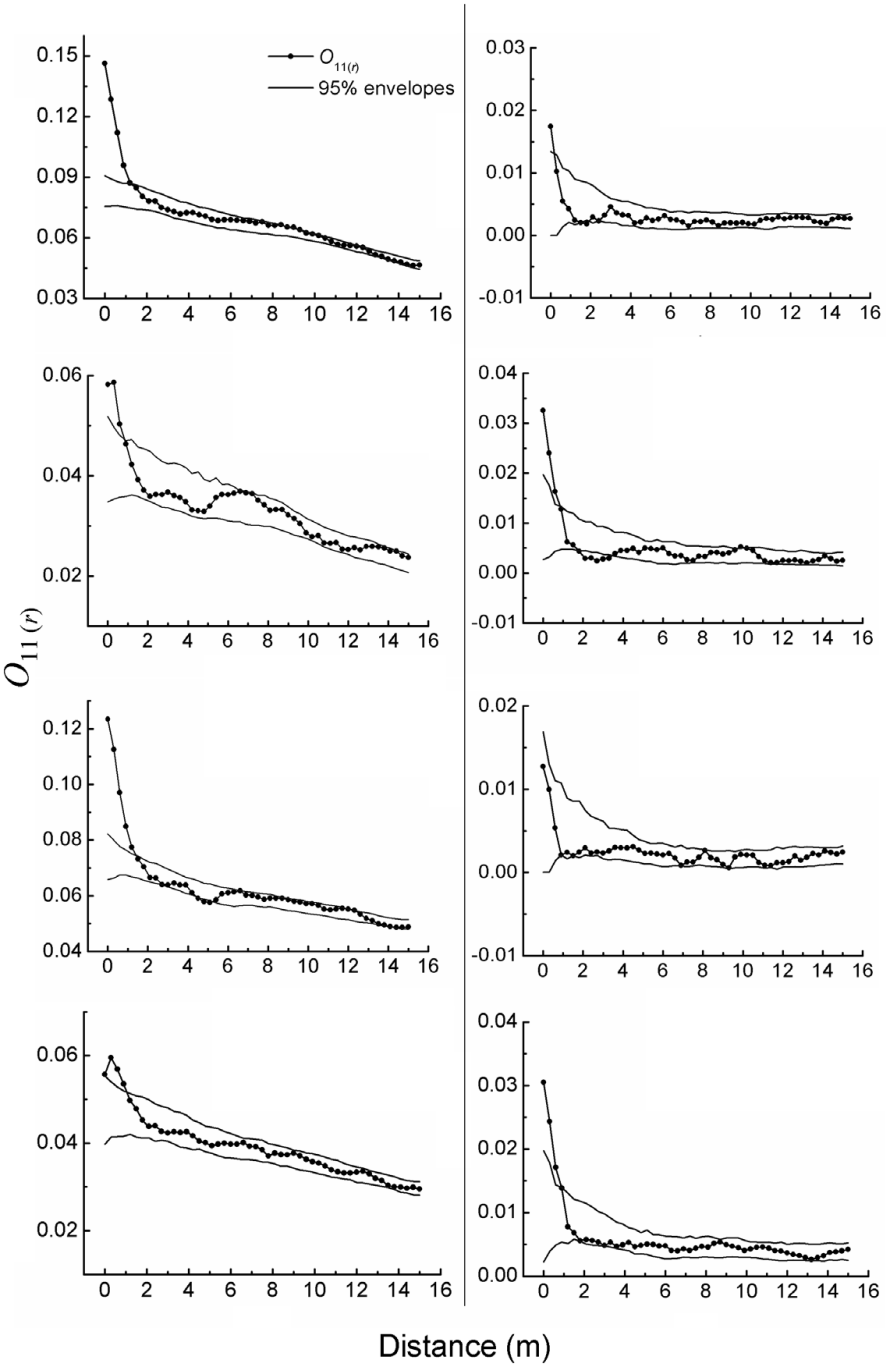
Spatial aggregation of *A. ordosica* saplings at the patch edges (left column, a) and the patch centers (right column, b), one sub-figure per plot.

Saplings at the patch edges were significantly aggregated at distances <0.6–1.2 m (Figure 2a). Saplings at the patch centers were significantly aggregated at distances <0.9 m (Figure 2b). The degrees of aggregation tended to weaken with increasing distances, and aggregation was more pronounced at the patch edges than at the patch centers.

**Figure 3:**
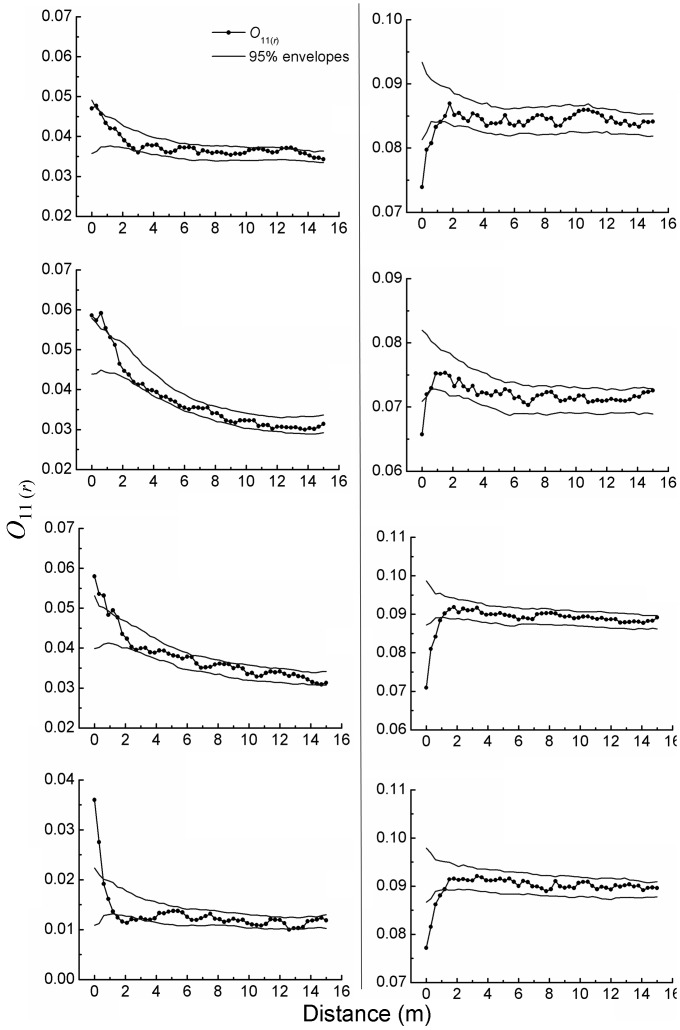
Spatial aggregation of *A. ordosica* adults at the patch edges (left column, a) and the patch centers (right column, b), one sub-figure per plot.

Adults at the patch edges were often significantly aggregated at distances <0.3–1.2 m (Figure 3a). This pattern also tended to weaken with increasing distances. Adults at the patch centers exhibited nearly random distribution at larger distances, similar to the prior cases, but distinctively overdispersion at a distance <0.3–0.9 m (Figure 3b). The adults were also aggregated more at the patch edges than at the patch centers.

Whether at the patch edges or at the patch centers, the saplings were more aggregative than the adults, mainly taking place at small distances of a couple of meters (Figures 2 & 3).

### Spatial Association of Saplings with Adults

**Figure 4:**
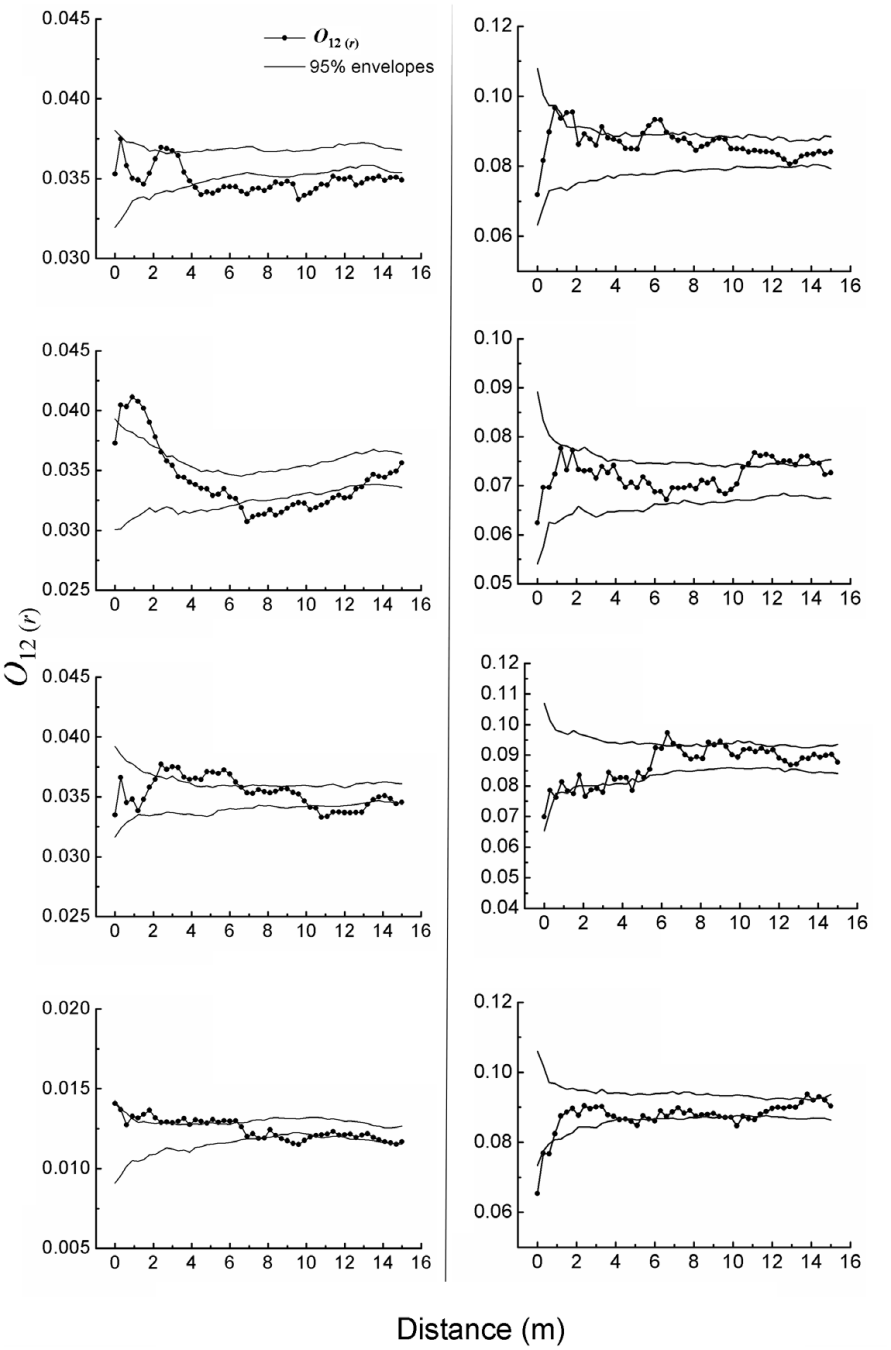
Spatial association of *A. ordosica* saplings with adults at the patch edges (left column, a) and the patch centers (right column, b), one sub-figure per plot.

At the patch edges, values of *O_12_*_(*r*)_ were above the upper envelopes at some distances, often within the range 0.3–6.6 m (Figure 4a). At other distances, the pattern decayed such that values of *O_12_*_(*r*)_ might decrease below the lower envelopes. These results indicate a mainly positive spatial association between saplings and adults at certain distances within the range 0.3–6.6 m, and a neutral and even negative association at other distances.

At the patch centers, values of *O_12_*_(*r*)_ mostly fell between the upper and lower envelopes, unlike those of the patch edges (Figure 4b). These results indicate that the spatial association of saplings with adults tended to be mostly random relative to adults, suggesting only a weak association between saplings and adults at the patch centers.

## Discussion

### Causes for Spatial Aggregation

Heterogeneous environmental conditions can cause aggregated plant spatial patterns, while homogeneous environments are unlikely to cause non-random patterns [32], [34], [35]. Nevertheless, plants that colonize homogeneous regions will affect surrounding conditions such as soil water, nutrients, or wind speeds, so as to affect mortality and patterns of neighbors and increase environmental heterogeneity [34], [36], [37]. Provided environments are originally homogeneous, interactions among plants will become primary causes to determine plant spatial patterns [37], [38]. Classic studies have reported that intense competition among plants can cause regular or uniform distribution by removing failures [32], [33], [39]. Contrarily, facilitation can cause aggregative distribution by enhancing viability of neighbors [32], [36], [40]. Neutral interactions can result in random distributions, which may also result from a net balance between competition and facilitation [37], [41].

Saplings of *A. ordosica* were more spatially aggregated than adults, and both were more aggregated at patch edges than at patch centers. Such patterns can reflect how they spatially organized and interacted to survive semi-arid sandy environments.

In semi-arid sandy land, soil moisture and sand drift are the predominant factors that influence plant survival [4], [23], [42]. Given the low precipitation, soil moisture in the Mu Us is usually low, thereby limiting the carrying capacity of the land for shrubs, i.e. the saturation point. From July to November, the volumetric water content varies around 11.5% in the 0–200 cm depth of the mobile sand [43]. Deficient moisture can cause the death of *A. ordosica* individuals, so that successful competition for moisture is crucial to their survival, and this further influences their spatial patterns [44], [45], [46]. Water competition was supposed to be more intense in areas with denser larger-crowned *A. ordosica* individuals, where soil moisture was already consumed greatly by the plants. From July to November, the volumetric water content varies around 5.4% in the 0–200 cm depth of the shrub-stabilized sand, thereby potentially causing higher mortality of *A. ordosica* individuals [43]. Thus, water competition may have inhibited aggregation of *A. ordosica* individuals by killing failures. Furthermore, shrub survival is adversely affected by sand drift that can blow, abrade, bury, and uproot *A. ordosica* individuals, especially more vulnerable saplings [4], [21], [23]. Even so, *A. ordosica* individuals can slow wind speeds with their standing branches and prevent sand from drifting [13], [47]. Due to differences in sizes, adult shrubs should be more effective at doing so than saplings. Spatially aggregated shrubs can reduce sand drifting and provide mutual protection for one another, but dispersed shrubs are more susceptible to sand drift and may have a higher mortality as a result [4], [23], [36]. Thus, sand drift might have maintained a higher degree of aggregation by removing some dispersed individuals. Other inferior factors, such as grasses, litter, ground crusts, and stochastic processes might also play some roles in shrub pattern formation.

A sapling with a smaller crown usually requires less soil water than an adult with a larger crown. Generally speaking, if saplings and adults are in the same density and moisture conditions, water competition among the saplings may well be much weaker than among the adults. This condition should be favorable for the aggregation of saplings [33], [44]. On the other hand, saplings exhibit worse resistance to sand drift than adults. Sand drift could kill dispersed saplings more easily than dispersed adults [23]. We suppose that the greater aggregation of saplings compared with that of adults may have resulted from their weaker competition for water and worse resistance to sand drift. Seeds of *A. ordosica* are very light, with 1000-seed-weight of ca. 0.311 g [26]. Such seeds can be easily carried miles away from parent plants, flying in the air or continually drifting on the bare ground with winds. Distances of seed dispersal are usually much larger than hundreds of meters, so seed dispersal is unlikely to have contributed much to the patterns we observed within tens of meters.

At the patch edges, where *A. ordosica* coverage was much lower and soil moisture was relatively higher, competition for water was likely weaker [43], [48]. However, sand drift was more intense and frequent due to worse protections provided by lower-coverage vegetation [47], [49]. Oppositely, at the patch centers with higher *A. ordosica* coverage, water competition was likely much stronger and sand drift was less intense [43], [47], [49]. These conditions might have induced stronger aggregation of *A. ordosica* individuals at the patch edges and weaker aggregation at the patch centers. At the patch edges, dispersed shrubs might die from sand drifting more easily than aggregated ones; at the patch centers, aggregated shrubs were more likely to die from water competition than dispersed ones. This reflects ecological adjustment of the shrubs from facilitation states to competition states [50].

### Dependence of Saplings on Adults

At the patch edges, spatial association of saplings with adults was mainly positive at certain distances between 0.3 and 6.6 m, and became neutral or even negative at other distances. These results suggest that saplings were more likely to survive if their distances from adults were 0.3–6.6 m. This presumably involved water competition and sand drift as well. All shrubs must consume soil water and also stabilize sand, more or less [47], [51]. When a sapling was farther from an adult, it would face weaker water competition but might experience more pronounced effects of sand drift. When saplings were 0.3–6.6 m away from an adult, they could optimally avoid the strongest water competition with the adult and also benefit from sand stabilization of the adult [23], [39], [52]. Thus, they were more likely to survive, leading to values of *O_12_*_(*r*)_ often higher than the upper envelopes (Figure 4a). Saplings located less than the optimal distances might have been less likely to survive because of intolerable water-competition with the adult, leading to values of *O_12_*_(*r*)_ lower than the upper envelopes (Figure 4a) [39]. Saplings that were>the optimal distances probably had a lower chance of survival because stronger sand drift could also kill them, leading to values of *O_12_*_(*r*)_ lower than the upper or even lower envelopes (Figure 4a) [4], [23].

At the patch centers, the saplings were associated almost neutrally with the adults; that is, their distances from adults had no significant effects on where they appeared. Water competition and sand drift were inferred to have an influence also on this pattern. At the patch centers, *A. ordosica* coverage almost reached their maximum, a saturation point that corresponds to precipitation [48]. Meanwhile, their spatial distribution became fairly uniform or homogeneous, as demonstrated above (Figures 2b & 3b). Homogeneous plant pattern often induces or associates with homogeneous environmental conditions [27], [43], [51]. Thus, soil moisture should have become homogenized throughout the patch centers, and likewise water competition. On the other hand, sand drift became very weak as well as homogenized after *A. ordosica* coverage reached the maximum and became spatially homogeneous [25], [47]. We assume that such increased homogeneity caused the almost random pattern of saplings relative to adults at the patch centers, i.e., neutral association of saplings with adults.

Saplings are recruits to a population. Thus, we conclude that patterns of *A. ordosica* adults are important for the species to recruit, and their effects are dependent on the balance between negative water-competition and positive sand-stabilization [41], [53].

### Population Recruitment and Landscape Recovery

The above results demonstrated that *A. ordosica* saplings were of higher proportion at the patch edges than at the patch centers. This meant that population recruitment was better at the patch edges than at the patch centers, presumably related to environmental differences between the patch edges and the patch centers.

At the patch centers, *A. ordosica* individuals were fully established, with the coverage of ca. 50%, nearly reaching its maximum [43], [48]. Given such conditions, colonization sites and water resources were likely available to new recruits only after some individuals died off [54]. Even if many seeds could germinate suddenly and grow as saplings or seedlings for some time, most of them would die from intense competition for water resources with surrounding denser larger-crowned adults, and only a few could happen to survive, as observed in the current study [43], [48].

At the patch edges, *A. ordosica* coverage was much lower than the maximum point, such that their competition for soil water probably remained weaker, and soil water and space were likely available for new recruits. Namely, patch edges should be favorable for sapling emergence and survival. In addition, sand drifts often become weakened under the protection of nearby standing adults. Such weakening has been shown to be advantageous also for sapling emergence and survival [23], [55].

We foresee that some saplings at patch edges will survive to adulthood due to better availability of soil water and proper weakening of sand drift, thus that edges will gradually merge into patch centers [23], [43], [49]. Outside old edges, new edges will reemerge as a result of seed supply as well as better conditions that enable them to thrive [26], [43], [49]. In this way, SSL patches that are covered by *A. ordosica* can expand stepwise towards the surrounding MSL patches, which accounts for the passive restoration of desertified landscapes. The results suggest that some native shrubs that grow in SSL patches in a semi-arid sandy land can act as competent engineers to promote passive restoration of surrounding MSL patches.

In semi-arid sandy land, we encourage local residents to protect SSL patches from further degradation, allowing them to smoothly expand outwards. A more active approach is that local residents artificially build up many size-suitable SSL patches with native shrubs in bare sand. If so, the process of passive restoration is artificially initiated and hastened. Such patches can also spontaneously expand outwards to revegetate bare sand. There may be many native shrub-species in a sandy area, and the ones whose seeds adapt to wind-dispersing may be preferable, like *A. ordosica* of the Mu Us which can disperse easily from parent shrubs with winds [13], [26], [56].
